# Effect of Fermented Mulberry Leaves on Gut Health of Finishing Pigs

**DOI:** 10.3390/ani14192911

**Published:** 2024-10-09

**Authors:** Su Peng, Yiyan Cui, Miao Yu, Min Song, Zhimei Tian, Dun Deng, Zhichang Liu, Xianyong Ma

**Affiliations:** 1 Institute of Animal Science, Guangdong Academy of Agricultural Sciences, Guangzhou 510640, China; pengsu222@163.com (S.P.); cuiyiyan@gdaas.cn (Y.C.); yumiao@gdaas.cn (M.Y.); songmin@gdaas.cn (M.S.); tianzhimei@gdaas.cn (Z.T.); dengdun@gdaas.cn (D.D.); 2 State Key Laboratory of Swine and Poultry Breeding Industry, Guangzhou 510640, China; 3 Key Laboratory of Animal Nutrition and Feed Science in South China, Ministry of Agriculture and Rural Affairs, Guangzhou 510640, China; 4 Guangdong Provincial Key Laboratory of Animal Breeding and Nutrition, Guangzhou 510640, China

**Keywords:** fattening pigs, fermented mulberry leaves, gut health, nutrient digestibility

## Abstract

**Simple Summary:**

Mulberry leaves have high nutritional and bioactive substance contents and are considered an alternate livestock feeding resource in China. However, due to the high content of antinutritional factors (e.g., tannin), the use of mulberry leaves is limited in animal production. Microbial fermentation reduces the content of antinutritional substances and increases the content of probiotics and bioactive constituents. Therefore, we fed finishing pigs 10% fermented mulberry leaves, which improved the digestion and absorption of nutrients, intestinal morphology, antioxidant ability, and immunity to enhance the gut health of finishing pigs.

**Abstract:**

This study was conducted to investigate the effects of supplementing fermented mulberry leaves (FML) on intestinal morphology, antioxidant capacity, and immune function in the gut of finishing pigs. Eighteen 132-day-old healthy crossbred (Duroc × Landrace × Yorkshire) male castrated pigs were randomly divided into two treatment groups with nine replicates per group. The control (CON) group was fed the basal diet, and the FML group was fed the basal diet supplemented with 10% FML. The experiment lasted 69 days. The results showed that 10% FML improved gut health. The apparent total tract digestibility in dry matter, crude protein, crude fiber, neutral detergent fiber, acidic detergent fiber, ether extract, and crude ash increased in the 10% FML group of finishing pigs compared to the CON group (*p* < 0.05). Duodenal, jejunal, and ileal intestinal morphology, such as villus height and villus-height-to-crypt-depth ratio, increased in the 10% FML group compared to the CON group, whereas crypt depth decreased in the duodenum, jejunum, and ileum (*p* < 0.05). Total antioxidant capacity increased in the ileum of the 10% FML group compared with the CON group (*p* < 0.05). The FML supplementation improved the contents of duodenal immunoglobulin A, jejunal interleukin-1β, interleukin-8, ileal interleukin-1β, interleukin-6, interferon-γ, and immunoglobulins A and M compared to the control group (*p* < 0.05). Moreover, FML downregulated the mRNA expression levels of tumor necrosis factor-α in the duodenum, Toll-like receptor 4, nuclear factor-κ B-P65, and myeloid differentiation factor 88 in the jejunum, and Toll-like receptor 4 and nuclear factor-κ B-P65 in the ileum (*p* < 0.05). The FML also upregulated Montrose uniting church 1 in the duodenum and claudin 2 in the ileum (*p* < 0.05). In conclusion, dietary supplementation with 10% FML improved the gut health of finishing pigs and FML is a potential feed ingredient for pig breeding.

## 1. Introduction

Conventional feedstuffs comprise the highest percentage of total livestock production cost. Thus, searching for inexpensive and reasonable feed resources to reduce cost is a research hotspot [1,2].

Mulberry trees are widely distributed throughout China. Mulberry leaves have higher protein content (14.0–34.2%) than other traditional forages, and several studies have reported that the active substances (e.g., polyphenols, polysaccharides, and alkaloids) have antioxidation, immune regulation, antistress, lipid-lowering, and other biological functions to improve growth performance, antioxidant capacity, and immune function in animals [3,4,5,6,7,8]. Because of their high nutritional value and bioactive substance contents, mulberry leaves are considered an alternate livestock feeding resource in animal husbandry [3,4,9]. As tannins can affect palatability, reduce the digestion and absorption rate of nutrients, and destroy digestive tract structure, excess tannins have an inhibitory effect on normal physiological activity and the basic metabolism of animals [10,11,12,13]. The high levels of tannins in mulberry leaves limit their application in animal husbandry. Therefore, to mitigate the negative effects of antinutritional factors on the intestinal tract and increase the amounts of mulberry leaves added for pig production [1], we fermented mulberry leaves (FML), and tannin content decreased by 56.4% [14], which improved the feeding value of the mulberry leaves.

Researchers have proposed that adding 4%, 5%, 12%, and 15% mulberry leaves reduces feed conversion rates in finishing pigs [3,6,8,15]. Furthermore, fermented mulberry leaves are more easily digested and absorbed than unfermented mulberry leaves [1]. Adding 25.5% FML increases the feed intake of sows and the weaning weights of 21-day-old piglets and reduces body weight loss in sows [16]. Another study showed that 10% and 20% FML-supplemented diets resulted in superior digestibility and increased the final body weight of broilers [2]. At present, FML are rarely used in fattening pig production.

Based on the previous results of our team, fermentation reduced the tannin content in mulberry leaves by 56.4% and increased the crude protein content by 16.8% [14]. However, very few studies have investigated the effects of FML in finishing pigs. Gut health is essential for digestion and absorption of nutrients as well as homeostasis [17,18]. Inflammatory responses in the intestine can result in intestinal damage, decreased nutrient absorption, and heightened susceptibility to diseases. Meanwhile, antioxidant activity functions to protect the intestinal mucosa from oxidative damage. Therefore, the objective of this study was to investigate the effect of dietary supplementation with 10% FML on gut health in finishing pigs, which will provide a reference for applying FML during pig production.

## 2. Materials and Methods

### 2.1. Preparation and Fermentation of the Mulberry Leaves

The mulberry leaves were provided by the Sericulture and Agro-Food Research Institute of Guangdong Academy of Agriculture Sciences (Guangzhou, Guangdong, China). The conditions for preparing the FML have been described previously [14]. Fresh mulberry leaves, with a moisture content of 68%, were cut to 1–2 cm and mixed with unsterilized wheat bran as the fermentation substrate at a 9:1 mass ratio. Then, 2% *Pediococcus cellicola* and *Bacillus licheniformis* were added at the concentration of 1.5 × 10^6^ CFU/mL to the substrate and stirred. The fermentation substrate was not sterilized. It was sealed at the natural pH value without any adjustment and a temperature of 25 ± 1 °C for 4 days. The nutritional levels of the FML are shown in Table 1 [19].

### 2.2. Animal Experimental Design

Eighteen healthy crossbred (Duroc × Landrace × Yorkshire) male castrated pigs (age 132 days), with an average body weight of 78.2 ± 2.1 kg, were balanced for initial body weight and randomly divided into two treatment groups. Each treatment group had nine replicates of one pig per replicate pen based on initial body weight. All animals were housed in 18 clean and disinfected separate pens (1.6 m × 4.7 m) equipped with slatted floors in the same house for 69 days during the experiment and had free access to food and water. Pigs in the control (CON) group were fed the basal diet, and the FML group was fed the basal diet supplemented with 10% FML. The feed was iso-energetic and iso-nitrogenous (Table 2) [19]. Changes in other dietary components were required to ensure that 10% FML could replace part of the corn and soybean meal and meet the growth requirements of the fattening pigs. This ultimately led to changes in the amount of corn and soybean meal added. The experiment was conducted at the pig farm of the Institute of Animal Science, Guangdong Academy of Agricultural Sciences. The animal procedures and experiments were approved by the Animal Care and Use Committee of the Guangdong Academy of Agricultural Sciences (authorization number GAASIAS-2021-0909).

### 2.3. Sample Collection

Three days before the end of the trial, fresh fecal samples were collected from all pigs via rectal massage. After fully mixing the fecal samples of each pen of pigs, 300 g was taken, the hair was removed, and 10 mL of 10% hydrochloric acid was added to mix thoroughly. For each fecal sample, there were three duplicates. The fecal samples were dried in a forced-air-drying oven at 65 °C for 72 h and then pulverized and stored at −80 °C until further chemical analysis [20].

At the end of the feeding period (day 69), 6 replicates in each group were randomly selected and euthanized by electric shock after fasting for approximately 12 h. Samples from the middle of the duodenum, jejunum, and ileum (3 cm) were collected in 4% paraformaldehyde for morphological analyses. The other parts of the duodenum, jejunum, and ileum were collected in 2 mL cryopreservation tubes and stored at −80 °C to determine antioxidant and immune indicators as well as for the mRNA expression analysis.

### 2.4. Apparent Total Tract Digestibility

Powdered feces and feed samples (2 g) were analyzed for nutrients and dry matter (DM) by drying in an electric blast-drying oven for 5 h. Crude protein (CP) was analyzed with an Automatic Nitrogen Analyzer (FOSS, Kjeltec™ 8400, Höganäs, Sweden) using the Kjeldahl method. An ether extract (EE) was measured using a fat filter bag and a 1 g sample on an automatic fat instrument (Ankom, XT15i, Macedon, NY, USA) and the result was calculated using a formula according to sample weight. Crude ash was measured in 1.5 g samples with the SX2-4-10N Box Resistance Furnace (Yiheng, Shanghai, China) for 5 h at 550 °C. Crude fiber (CF), acid detergent fiber (ADF), and neutral detergent fiber (NDF) were determined with a semi-automatic fiber analyzer (Ankom Technology, A200, Macedon, NY, USA) using the filter bag method. All of these factors were determined by previously described methods [21].

Apparent total tract digestibility (ATTD) was determined by the acid-insoluble ash (AIA) method [22]. This method decomposes the organic matter in the sample through ashing. The ash obtained is treated with hydrochloric acid and the ash in the sample that is insoluble in dilute hydrochloric acid is AIA. The formula is:

ATTD (%) = [1 − (N_feces_ × AIA_diet_)/(N_diet_ × AIA_feces_)] × 100

where N_feces_ represents the content of each nutrient in the fecal sample, AIA_diet_ is the nutrient AIA content in the diet, N_diet_ is the content of each nutrient in the diet, and AIA_feces_ is the nutrient AIA content in a fecal sample. The nutrients included DM, CP, EE, crude ash, CF, ADF, and NDF.

### 2.5. Intestinal Morphology

Fixed intestinal rings were paraffin embedded, sectioned, stained with hematoxylin and eosin, and sealed. We used the digital slice scanning software Case Viewer 2.4 to measure intestinal villus height (VH) and crypt depth (CD) in the groups. We also analyzed the VH, CD, and the villus-height-to-crypt-depth ratio (VH:CD) of the intestine.

### 2.6. Antioxidant Capacity

The antioxidant parameters, including malondialdehyde (MDA), total antioxidant capacity (T-AOC), glutathione peroxide (GSH-Px), and total superoxide dismutase (T-SOD) in the duodenum, jejunum, and ileum were tested in duplicate using kits from Nanjing Jiancheng Bioengineering Institute (Nanjing, China), following the manufacturer’s instructions.

### 2.7. Intestinal Immunity

The contents of interleukin (IL)-1β, IL-2, IL-6, IL-8, IL-10, IL-22, tumor necrosis factor (TNF)-α, interferon (INF)-γ, and immunoglobulins (IgA, IgG, and IgM) in the duodenum, jejunum, and ileum were determined using enzyme-linked immunosorbent assay kits from Shanghai Enzyme-linked Biotechnology Co., Ltd. (Shanghai, China).

### 2.8. RNA Extraction and cDNA Synthesis

Total RNA was extracted from the duodenum, jejunum, and ileum using Trizol regent (Takara, Dalian, China) according to the manufacturer’s instructions. A NanoDrop 1000 spectrophotometer (Thermo Fisher Scientific Inc., Wilmington, DE, USA) was used to quantify the RNA concentration and purity of the samples. The RNA OD_260_:OD_280_ ratio ranged from 1.8 to 2.0. Approximately 1.0 μg of total RNA sample was reverse transcribed to cDNA using an mRNA reverse transcription kit (Takara) following the manufacturer’s instructions.

### 2.9. Real-Time Quantitative Polymerase Chain Reaction (PCR) Analysis

Real-time quantitative PCR (RT-qPCR) analysis of the target genes and the housekeeping gene ribosomal protein L-4 (RPL4) was conducted on a CFX96 Real-Time qPCR Detection system (Bio-Rad, Hercules, CA, USA) using TB Green™ Premix Ex Taq™ (Takara). The specific primer sequences of the target genes are presented in Table 3. The RT-qPCR system (total of 20 μL) consisted of 10.0 μL of TB Green™ Premix Ex Taq™, 2.0 μL of template cDNA, 7.2 μL of double-distilled water, and 0.4 μL of the upstream and 0.4 μL of the downstream primers. The reaction mixtures and real-time qPCR conditions were the same as those used in a previous study [23]. RPL4 was chosen as the endogenous housekeeping gene to express the levels of each target gene. The RT-qPCR cycling conditions were: 95 °C for 30 s, repeated for 40 cycles at 95 °C for 5 s, and 60 °C for 30 s in a denaturation program, followed by a 60–95 °C increase at a heating rate of 0.1 °C/s under melting curve conditions. The melting curves were verified to ensure single-product amplification at a consistent melting temperature after amplification. Quantification was performed in duplicate. The average cycle threshold (Ct) value for each sample was calculated according to the formula 2^−(ΔΔCt)^, where ΔΔCt  =  (Ct_target_ − Ct_β-actin_) _treatment_ − (Ct_target_ − Ct_β-actin_) _control_ [24].

### 2.10. Statistical Analysis

All experimental data were analyzed using IBM SPSS Statistics 26.0 software (IBM Corp., Armonk, NY, USA) and were subjected to the independent *t*-test. The normality of the data was judged by the Shapiro–Wilk test before assessing differences between the groups. Variability in the data is expressed as mean ± standard error. A *p*-value < 0.05 was considered significant, 0.05 < *p*-value < 0.10 was considered tending.

## 3. Results

### 3.1. Apparent Total Tract Digestibility

The effects of FML on the nutrient digestibility of finishing pigs are shown in Table 4. Fermented mulberry leaves increased ATTD of DM (*p* = 0.021), CP (*p* = 0.038), CF (*p* = 0.024), NDF (*p* = 0.005), ADF (*p* = 0.007), EE (*p* = 0.007), and ash (*p* = 0.001) compared to the CON group.

### 3.2. Intestinal Morphology

Pigs fed the FML diets had greater increased VH (*p* < 0.05) and VH:CD (*p* < 0.001) and decreased CD (*p* < 0.001) in the duodenum, jejunum, and ileum than pigs fed the basal diet (Table 5).

**Figure 1:**
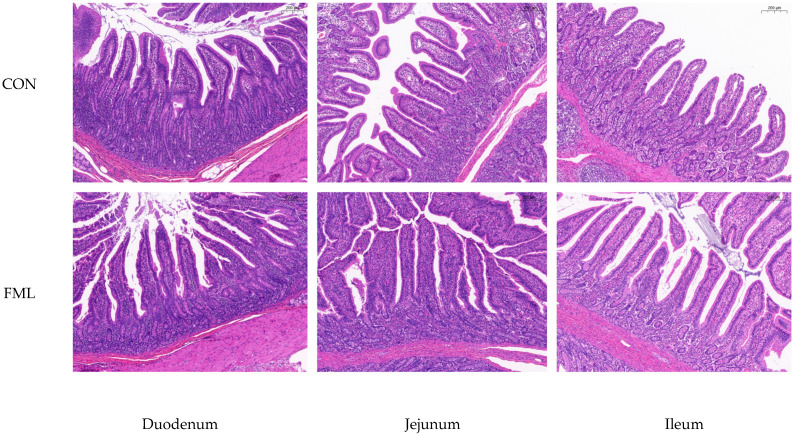
The small intestinal morphology in finishing pigs. CON, fed with the basal diet; FML, fed with the basal diet supplemented with 10% fermented mulberry leaves.

As can be observed from Figure 1, the morphology of small intestinal villi in fattening pigs fed with FML has undergone significant changes compared to the control group. The length has increased, the arrangement is orderly, providing a guarantee for the efficient absorption of nutrients.

### 3.3. Antioxidant Capacity

The antioxidant capacity of the duodenum, jejunum, and ileum is presented in Table 6. Dietary FML increased T-AOC (*p* = 0.009) in the ileum and tended to increase GSH-Px activity (*p* = 0.062) in the jejunum.

### 3.4. Intestinal Tissue Immunity

As shown in Table 7, dietary FML increased the content of duodenal IgA (*p* = 0.041), jejunal IL-1β (*p* = 0.023) and IL-8 (*p* = 0.040), and ileal IL-1β (*p* = 0.002), IL-6 (*p* = 0.003), INF-γ (*p* = 0.004), IgA (*p* < 0.001), and IgM (*p* = 0.040). Moreover, FML supplementation tended to increase the contents of duodenal IL-1β (*p* = 0.096) and TNF-α (*p* = 0.097) and jejunal IL-6 (*p* = 0.099) and IgM (*p* = 0.081).

### 3.5. Gene Expression

As shown in Table 8, FML decreased the expression of TNF-α in the duodenum (*p* = 0.038), increased the expression of MUC 1 (*p* < 0.001), and tended to increase the expression of IGF1R (*p* = 0.064) compared to the CON group. The FML downregulated the mRNA expression levels of TLR4 (*p* = 0.020), NFκB-P65 (*p* = 0.009), and MyD88 (*p* = 0.048) in the jejunum and tended to upregulate the IL-10 expression level (*p* = 0.063). The FML diet downregulated the expression levels of TLR4 (*p* = 0.046), NFκB-P65 (*p* = 0.026), and MyD88 (*p* = 0.061) and tended to upregulate the expression levels of claudin 1 (*p* = 0.069) and claudin 2 (*p* = 0.036) in the ileum.

## 4. Discussion

More attention is being paid to the effect of alternative and locally available feedstuffs on pig breeding, as the cost of conventional feedstuffs has increased [6]. An important index to evaluate the digestibility of dietary nutrients is apparent nutrient digestibility. Improving nutrient digestibility and reducing nutrient excretion are crucial for enhancing animal production performance and promoting sustainable breeding. Previous research has reported that supplementing mulberry leaf pellets, FML, and mulberry leaf flavonoids increases apparent digestibility in beef cattle, broilers, and sheep, respectively [1,25,26]. Previous research has also demonstrated that dietary DM, CP, and CF digestibility in the FML group of broilers was better than that of unfermented mulberry leaves [1,2]. Therefore, we first examined whether 10% FML in the diet improved nutrient digestibility in finishing pigs. As results, consuming FML increased the digestibility of DM, CP, CF, NDF, ADF, EE, and ash by 4.68%, 5.18%, 43.89%, 44.88%, 38.72%, 159.92%, and 81.62%, respectively, compared with the CON group, which was similar to previous studies [1,2,25,26,27]. The inclusion of 10% FML in the diet has a positive impact on the finishing pigs’ ability to digest and absorb nutrients. It may be that the microorganisms in fermented mulberry leaves transform mulberry leaf components into microbial proteins, active peptides, and other active substances. This reduces the content of antinutritional factors and improves the digestibility and absorption rate of nutrients [28]. The highly active cellulase and amylase generated by *Pediococcus cellicola* and *Bacillus licheniformis* are capable of degrading fiber and carbohydrates [29]. Finishing pigs supplemented with FML had a better ability to digest fiber and enhanced their coarse feeding tolerance. The reason may be that mulberry leaves are fermented by microorganisms, which highlights the advantages of each strain. *Pediococcus cellicola* and *Bacillus licheniformis* have a synergistic effect with the original microorganisms in the gut. In addition, FML improves intestinal morphology, and the higher VH and the lower CD in the small intestine, where most digestion and absorption of nutrients occur in pigs, suggest a larger surface area and greater absorption of nutrients [30], while secreting various digestive enzymes [31]. In the current study, FML supplementation may have improved intestinal absorption due to greater VH in the small gut and the increase in epithelial turnover of the nutrients due to an increased VH:CD of the duodenum, jejunum, and ileum. Similar to our results, broilers fed a diet supplemented with FML powder had increased VH:CD of the duodenum, jejunum, and ileum and improved activity of intestinal digestive enzymes [1]. A mulberry leaf extract enhances the development of the foregut in the giant salamander [27] and increases VH and the VH:CD of the jejunum and ileum in weaned piglets [32]. Some reports suggest that the influence on digestive enzyme activity might be associated with the active components in mulberry leaf extract [27,33]. In addition, probiotics produce lactic acid, digestive enzymes, and other substances by fermenting in the intestine and promote the digestion and absorption of nutrients [4,26,34,35]. Therefore, dietary FML promotes bioavailability by improving intestinal histomorphology. The promoting effect of the FML may also be related to an improved small intestinal environment and the microbial composition due to active substances and the beneficial fermentation bacteria in FML. However, the specific mechanism needs to be further explored. Replacing part of the soybean meal and corn with FML increased the digestibility of nutrients in finishing pigs, reduced the cost of feeding, and enhanced intestinal health.

The intestinal tract is where the body digests and absorbs nutrients from food and is the most vulnerable to free radicals in animals [6,36,37,38,39,40]. The antioxidant system prevents damage caused by lipid and protein oxidation, maintains the integrity of the intestinal barrier, and prevents bacterial infection [41]. T-AOC, SOD, MDA, and GSH-Px are important antioxidant indicators reflecting the antioxidant capacity of the body [42,43,44]. Mulberry leaves are a source of natural antioxidants due to the contents of various secondary metabolites (flavonoids, alkaloids, and polysaccharides) with antioxidant activity [4,9,33,45]. Supplementing with mulberry leaves increases the antioxidant capacity of cattle, sheep, and laying hens [4,9,46]. Our previous study showed that FML did not improve antioxidant enzyme activities in the gut but increased blood glutathione content [19]. The antioxidant enzyme activities in the small intestine and blood were not affected by FML.

Intestinal health is also reflected by cytokine levels. Normal cytokine levels increase anti-inflammatory activity [47], but higher levels activate the inflammatory pathway [48,49,50,51], and an excessive inflammatory reaction decreases digestive function [38,52]. A previous report has shown that mulberry leaf polysaccharides increase cytokine (IL-1β, IL-2, IL-6, IL-8, and IFN-γ) contents and improve piglet immunity [7]. A mulberry leaf extract downregulated the transcription factor NF-κB to exert anti-inflammatory activity [4]. Similar to the results of previous studies, small intestinal cytokines, such as IL-1β, IL-6, IL-8, and IFN-γ, increased in the FML group. IL-1β is involved in the regulation of tissue damage [7,53], IL-6 exhibits anti-inflammatory effects [7], IL-8 enhances the adaptive immune response [50], TNF-α exhibits an essential role in host defense and exerts an antibacterial effect by activating macrophages [48,49], and IFN-γ is involved in the initiation and regulation of the immune response [7]. FML could play an immunomodulatory role, as the active substance in FML may prime phagocytes for a significantly enhanced anti-inflammatory response by regulating cytokine content. Additionally, TLR4 is expressed at higher levels in intestinal epithelial cells of an inflamed bowel [54]. It stimulates and enhances activation of the signaling molecules MyD88 and NF-κB, forming the MyD88, NF-κB, and TLR4 inflammatory pathway, which is harmful to the body [48,49,50,51,55]. The MyD88, NFκB-P65, and TLR4 mRNA expression levels decreased, further illustrating that cytokine content increased within the normal range in our study because they did not over-stimulate the reaction with positive effects on digesting and absorbing nutrients in the FML group, and this means FML was beneficial to intestinal immune health. The vast surface area of the intestine is constantly exposed to pathogenic microorganisms, but also a diverse milieu of antigenic dietary components, that facilitate the need for a sophisticated intestinal immune system [56]. Immunoglobulins, which are universally present in the body, are important indicators of humoral immunity that promote phagocytosis of macrophages and combine with antigens to produce a variety of biological effects [7,41]. Our findings indicate that FML induced an increase in small intestinal IgA and IgM, and our previous study indicated that FML induces increases in plasma IgA, IgG, and IgM [19], which may improve macrophage immunomodulatory activity [47]. The intestinal barrier protects against pathogenic agents and luminal antigens. Mucins are the main components of the intestinal mucus layer and play an important role in maintaining intestinal health. Studies have demonstrated that insufficient mucus secretion leads to susceptibility to intestinal inflammation and infection [57]. In the current study, FML upregulated the expression of intestinal barrier genes in the duodenum (MUC 1) and ileum (claudin 1 and claudin 2), suggesting that FML enhances the integrity of the mucous layer and generates a host-friendly gut environment to defend against pathogen infection.

To the best of our knowledge, information on the effect of mulberry leaves on the mRNA expression levels of cytokines, tight junction proteins, and mucin-related genes in the duodenum, jejunum, and ileum is very limited. Thus, high levels of small intestinal cytokines, immunoglobulins, barrier gene expression, and downregulated gene expression levels of MyD88, NFκB-P65, and TLR4 in the FML groups of finishing pigs revealed improved cellular and humoral immunity. This improved immunity was attributed to the enrichment of active substances and beneficial fermentation bacteria, as well as the inhibition of potentially harmful bacteria. Further studies are required to clarify the key active substances, the abundance or counts of beneficial fermentation bacteria, and the underlying mechanisms. In general, disrupting the intestine increases permeability which compromises gut barrier function, impairs digestibility and absorption of nutrients, and triggers inflammation [38]. However, as seen from our results, the use of FML as a supplement had beneficial effects on intestinal digestion and absorption, intestinal morphology, intestinal antioxidant activity, and immunity. These findings indicate that FML is useful as a green and healthy feeding resource for finishing pigs.

## 5. Conclusions

A 10% dietary supplement of FML improved intestinal morphology, such as VH, CD, and the VH:CD of the small intestine, which may have been related to improved digestion and absorption of nutrients, such as DM, CP, CF, NDF, ADF, EE, and ash. In addition, 10% FML promoted antioxidant activities, immunity, and intestinal mRNA expression levels in finishing pigs. Overall, including 10% FML in the diet created a healthy intestinal environment and improved the utilization of feed, resulting in a lower cost to fatten the pigs.

## Figures and Tables

**Table 1:** The nutrition levels of fermented mulberry leaves (FML) (DM basis) %.

Items ^1^	Moisture	CP	EE	CF	NDF	ADF	Ash	Ca	P	Tannin
FML	68.00	26.88	2.14	14.65	31.43	14.95	12.87	2.67	0.61	0.47
ML	68.75	22.41	1.57	13.80	38.25	14.65	12.7	2.72	0.53	1.09

^1^ ML, mulberry leaves; CP, crude protein; EE, ether extract; CF, crude fiber; NDF, neutral detergent fiber; ADF, acid detergent fiber; Ca, calcium; P, total phosphorus.

**Table 2:** Ingredients and nutrient levels of experimental diets (air-dried basis) %.

Items ^1^	CON Group	FML Group
Ingredients		
Corn	69.42	65.23
Soybean meal	16.49	11.67
Wheat bran	8.00	7.30
Soybean oil	2.60	2.60
L-lysine	0.38	0.41
L-methionine	0.05	0.12
L-threonine	0.06	0.08
L-tryptophan	0.01	0.05
CaHPO_4_	0.91	0.93
Limestone	0.78	0.31
NaCl	0.30	0.30
FML		10.00
Premix ^2^	1.00	1.00
Total	100.00	100.00
Nutrient levels ^3^		
Digestible energy, MJ/kg	14.21	14.49
Crude protein	15.21	15.18
Crude fiber	3.21	4.59
Calcium	0.61	0.68
Total phosphorus	0.50	0.52
Available phosphorus	0.23	0.22
Lysine	0.98	0.99
Methionine + cysteine	0.55	0.55
Threonine	0.59	0.59
Tryptophan	0.17	0.18

^1^ CON, control; FML, fermented mulberry leaves. ^2^ Premix provided the following per kg of diets: 6500 IU vA, 2000 IU vD_3_, 150 mg vE, 3 mg vK_3_, 0.03 mg vB_12_, 3 mg vB_1_, 6 mg vB_2_, 5 mg vB_6_, 45 mg nicotinic acid, 9 mg D-pantothenic acid, 1 mg folic acid, 0.3 mg biotin, 72 mg of Fe as FeSO_4_, 10 mg of Cu as CuSO_4_, 42 mg of Mn as MnSO_4_, 72 mg of Zn as ZnO, 0.42 mg I as ethylenediamine dihydroiodide, 0.2 mg of Se as Na2SeO_3_, 34 mg of Mg as MgO. ^3^ Crude protein and crude fiber were measured values, while the others were calculated values.

**Table 3:** Target Genes and Primer Sequences used for Quantification in this study.

Genes ^1^	Primer Name	Primer Sequence 5′–3′
RPL4	RPL4-F	GCTCTATGGCACTTGGCGT
	RPL4-R	GCGGAGGGCTCTTTGGAT
IL-8	IL-8-F	ACTGGCTGT TGCCTTCTT
	IL-8-R	CAGTTCTCTTCAAAAATATCTG
IL-10	IL-10-F	GTCCGACTCAACGAAGAAGG
	IL-10-R	GCCAGGAAGATCAGGCAATA
TLR4	TLR4-F	TCAGTTCTCACCTTCCTCCTG
	TLR4-R	GTTCATTCCTCACCCAGTCTTC
NFκB-P65	NFκB-P65-F	AACCCCTTCCAAGTTCCCA
	NFκB-P65-R	TCCCCGAGTTCCGATTCAC
MyD88	MyD88-F	TGCCTTCATCTGCTACTGC
	MyD88-R	GAGACAACCGCTACCATC
TNF-α	TNF-α-F	CCACGCTCTTCTGCCTACTGC
	TNF-α-R	GCTGTCCCTCGGCTTTGAC
IGF1R	IGF1R-F	GGGATGACGAGAGACATCTATGAG
	IGF1R-R	GAAGGACCAGACTCAGAGTGC
EGF	EGF-F	ATCTCAGGAATGGGAGTCAACC
	EGF-R	TCACTGGAGGATGGAATACAGC
Occludin	Occludin-F	ATGCTTTCTCAGCCAGCGTA
	Occludin-R	AAGGTTCCATAGCCTCGGTC
Claudin-1	Claudin-1-F	ATTTCAGGTCTGGCTATCTTAGTTGC
	Claudin-1-R	AGGGCCTTGGTGTTGGGTAA
Claudin-2	Claudin-2-F	GCATCATTTCCTCCCTGTT
	Claudin-2-R	TCTTGGCTTTGGGTGGTT
ZO-1	ZO-1-F	GAGGATGGTCACACCGTGGT
	ZO-1-R	GGAGGATGCTGTTGTCTCGG
MUC 1	MUC-1-F	GGTACCCGGCTGGGGCATTG
	MUC-1-R	GGTAGGCATCCCGGGTCGGA
MUC 2	MUC-2-F	CTGCTCCGGGTCCTGTGGGA
	MUC-2-R	CCCGCTGGCTGGTGCGATAC

^1^ RPL4, ribosomal protein L-4; IL-8, interleukin-8; IL-10, interleukin-10; TLR4, Toll-like receptor 4; NFκB-P65, nuclear factor-kappa B-P65; MyD88, myeloid differentiation factor 88; TNF-α, tumor necrosis factor-α; IGF1R, insulin-like growth factor 1 receptor; EGF, epidermal growth factor; ZO-1, zonula occludens-1; MUC 1, mucin 1; MUC 2, mucin 2.

**Table 4:** Effect of the FML on nutrient digestibility in finishing pigs ^1^.

Item ^2^	CON	FML	*p*-Value
Apparent total tract digestibility (%)			
DM	82.82 ± 1.18	86.70 ± 0.80	0.021
CP	81.90 ± 1.50	86.14 ± 0.58	0.038
CF	40.62 ± 5.59	58.45 ± 0.43	0.024
NDF	47.22 ± 4.62	68.41 ± 1.22	0.005
ADF	46.82 ± 4.33	64.95 ± 1.44	0.007
EE	18.54 ± 6.23	48.19 ± 2.09	0.007
Ash	30.52 ± 4.17	55.43 ± 1.42	0.001

^1^ CON, fed with the basal diet; FML, fed with the basal diet supplemented with 10% fermented mulberry leaves. Values are expressed as mean ± standard error, *n* = 6. ^2^ DM, dry matter; CP, crude protein; CF, crude fiber; NDF, neutral detergent fiber; ADF, acid detergent fiber; EE, ether extract.

**Table 5:** Effect of the FML on small intestinal morphology in finishing pigs ^1^.

Item ^2^	CON	FML	*p*-Value
Duodenum			
VH (μm)	593.03 ± 19.85	657.50 ± 19.78	0.024
CD (μm)	477.42 ± 11.09	340.32 ± 9.42	<0.001
VH:CD	1.12 ± 0.06	1.89 ± 0.16	<0.001
Jejunum			
VH (μm)	477.08 ± 14.53	647.01 ± 14.63	<0.001
CD (μm)	378.89 ± 9.44	282.69 ± 9.07	<0.001
VH:CD	1.12 ± 0.11	2.19 ± 0.10	<0.001
Ileum			
VH (μm)	421.79 ± 8.26	545.23 ± 9.63	<0.001
CD (μm)	315.17 ± 10.75	229.22 ± 7.09	<0.001
VH:CD	1.37 ± 0.06	2.44 ± 0.15	<0.001

^1^ CON, fed with the basal diet; FML, fed with the basal diet supplemented with 10% fermented mulberry leaves. Values are expressed as mean ± standard error, *n* = 6. ^2^ VH, villus height; CD, crypt depth; VH:CD, villus height to crypt depth.

**Table 6:** Effect of the FML on antioxidant capacity in finishing pigs ^1^.

Item ^2^	CON	FML	*p*-Value
Duodenum			
MDA (nmol/mgprot)	0.02 ± 0.01	0.02 ± 0.01	0.987
T-AOC (U/mgprot)	0.01 ± 0.00	0.01 ± 0.00	0.187
T-SOD (U/mgprot)	8.92 ± 0.43	9.94 ± 0.72	0.248
GSH-Px (U/mgprot)	28.57 ± 3.07	26.34 ± 3.06	0.618
Jejunum			
MDA (nmol/mgprot)	0.02 ± 0.00	0.03 ± 0.00	0.166
T-AOC (U/mgprot)	19.85 ± 1.15	21.26 ± 1.38	0.603
T-SOD (U/mgprot)	7.77 ± 0.85	10.00 ± 0.63	0.449
GSH-Px (U/mgprot)	0.02 ± 0.00	0.03 ± 0.00	0.062
Ileum			
MDA (nmol/mgprot)	0.02 ± 0.00	0.01 ± 0.00	0.144
T-AOC (U/mgprot)	0.01 ± 0.00	0.02 ± 0.00	0.009
T-SOD (U/mgprot)	8.76 ± 0.78	8.34 ± 0.51	0.661
GSH-Px (U/mgprot)	5.91 ± 0.70	6.48 ± 0.16	0.446

^1^ CON, fed with the basal diet; FML, fed with the basal diet supplemented with 10% fermented mulberry leaves. Values are expressed as mean ± standard error, *n* = 6. ^2^ MDA, malondialdehyde; T-AOC, total antioxidant capacity; T-SOD, total superoxide dismutase; GSH-Px, glutathione peroxide.

**Table 7:** Effect of the FML on intestinal immunity in finishing pigs ^1^.

Item ^2^	CON	FML	*p*-Value
Duodenum			
IL-1β (pg/mgprot)	5.29 ± 0.07	6.11 ± 0.40	0.096
IL-2 (pg/mgprot)	3.01 ± 0.09	3.16 ± 0.20	0.512
IL-6 (pg/mgprot)	8.63 ± 0.17	9.52 ± 0.53	0.145
IL-8 (pg/mgprot)	0.41 ± 0.01	0.42 ± 0.02	0.573
IL-10 (pg/mgprot)	1.40 ± 0.02	1.55 ± 0.09	0.140
IL-22 (pg/mgprot)	2.91 ± 0.07	2.99 ± 0.17	0.674
TNF-α (pg/mgprot)	0.39 ± 0.01	0.44 ± 0.02	0.097
INF-γ (pg/mgprot)	0.36 ± 0.01	0.40 ± 0.03	0.243
IgA (μg/mgprot)	6.28 ± 0.10	8.77 ± 0.91	0.041
IgG (μg/mgprot)	147.74 ± 1.43	153.00 ± 7.56	0.522
IgM (μg/mgprot)	184.26 ± 5.18	200.48 ± 10.90	0.209
Jejunum			
IL-1β (pg/mgprot)	8.86 ± 0.26	11.00 ± 0.67	0.023
IL-2 (pg/mgprot)	5.66 ± 0.36	6.42 ± 0.38	0.178
IL-6 (pg/mgprot)	9.41 ± 0.61	10.90 ± 0.54	0.099
IL-8 (pg/mgprot)	0.63 ± 0.03	0.73 ± 0.03	0.040
IL-10 (pg/mgprot)	2.87 ± 0.19	3.14 ± 0.17	0.315
IL-22 (pg/mgprot)	6.59 ± 0.46	6.84 ± 0.37	0.680
TNF-α (pg/mgprot)	1.29 ± 0.06	1.34 ± 0.08	0.598
INF-γ (pg/mgprot)	1.06 ± 0.08	1.17 ± 0.07	0.299
IgA (μg/mgprot)	11.73 ± 0.77	12.75 ± 0.66	0.337
IgG (μg/mgprot)	291.07 ± 19.94	322.79 ± 21.15	0.301
IgM (μg/mgprot)	301.34 ± 25.35	368.57 ± 23.65	0.081
Ileum			
IL-1β (pg/mgprot)	5.14 ± 0.09	6.00 ± 0.16	0.002
IL-2 (pg/mgprot)	3.06 ± 0.07	3.21 ± 0.19	0.498
IL-6 (pg/mgprot)	7.68 ± 0.13	8.41 ± 0.12	0.003
IL-8 (pg/mgprot)	0.41 ± 0.01	0.42 ± 0.01	0.468
IL-10 (pg/mgprot)	1.28 ± 0.31	1.69 ± 0.10	0.201
IL-22 (pg/mgprot)	2.71 ± 0.06	2.97 ± 0.17	0.222
TNF-α (pg/mgprot)	0.42 ± 0.02	0.46 ± 0.02	0.201
INF-γ (pg/mgprot)	0.35 ± 0.01	0.40 ± 0.01	0.004
IgA (μg/mgprot)	6.12 ± 0.09	9.28 ± 0.23	<0.001
IgG (μg/mgprot)	156.80 ± 10.06	159.73 ± 10.00	0.841
IgM (μg/mgprot)	163.51 ± 0.56	199.52 ± 11.76	0.040

^1^ CON, fed with the basal diet; FML, fed with the basal diet supplemented with 10% fermented mulberry leaves. Values are expressed as mean ± standard error, *n* = 6. ^2^ IL-1β, interleukin-1β; IL-2, interleukin-2; IL-6, interleukin-6; IL-8, interleukin-8; IL-10, interleukin-10 IL-22, interleukin-22; TNF-α, tumor necrosis factor-α; INF-γ, interferon-γ; IgA, immunoglobulins A; IgG, immunoglobulins G; IgM, immunoglobulins M.

**Table 8:** Effect of FML on the gene expression of intestinal tissue in finishing pigs ^1^.

Item ^2^	CON	FML	*p*-Value
Duodenum			
IL-8	1.00 ± 0.23	1.32 ± 0.11	0.267
IL-10	1.00 ± 0.14	1.16 ± 0.31	0.664
TLR4	1.00 ± 0.10	1.13 ± 0.10	0.406
NFκB-P65	1.00 ± 0.26	0.59 ± 0.22	0.287
MyD88	1.00 ± 0.35	0.71 ± 0.05	0.450
TNF-α	1.00 ± 0.11	0.61 ± 0.11	0.038
IGF1R	1.00 ± 0.25	2.00 ± 0.39	0.064
EGF	1.00 ± 0.45	1.56 ± 0.55	0.459
Occludin	1.00 ± 0.19	1.51 ± 0.30	0.185
Claudin 1	1.00 ± 0.26	0.70 ± 0.17	0.362
Claudin 2	1.00 ± 0.26	1.35 ± 0.18	0.353
ZO-1	1.00 ± 0.23	0.65 ± 0.35	0.427
MUC 1	1.00 ± 0.15	3.87 ± 0.23	<0.001
MUC 2	1.00 ± 0.31	1.35 ± 0.29	0.427
Jejunum			
IL-8	1.00 ± 0.29	0.91 ± 0.25	0.816
IL-10	1.00 ± 0.13	1.55 ± 0.25	0.063
TLR4	1.00 ± 0.19	0.36 ± 0.11	0.020
NFκB-P65	1.00 ± 0.14	0.28 ± 0.06	0.009
MyD88	1.00 ± 0.23	0.42 ± 0.14	0.048
TNF-α	1.00 ± 0.25	0.70 ± 0.19	0.408
IGF1R	1.00 ± 0.22	1.33 ± 0.21	0.323
EGF	1.00 ± 0.17	0.75 ± 0.29	0.465
Occludin	1.00 ± 0.22	0.66 ± 0.13	0.231
Claudin 1	1.00 ± 0.25	0.87 ± 0.19	0.688
Claudin 2	1.00 ± 0.17	1.16 ± 0.09	0.517
ZO-1	1.00 ± 0.61	0.20 ± 0.12	0.248
MUC 1	1.00 ± 0.15	0.56 ± 0.25	0.163
MUC 2	1.00 ± 0.16	0.65 ± 0.03	0.194
Ileum			
IL-8	1.00 ± 0.29	1.40 ± 0.09	0.264
IL-10	1.00 ± 0.23	1.16 ± 0.16	0.621
TLR4	1.00 ± 0.26	0.28 ± 0.07	0.046
NFκB-P65	1.00 ± 0.12	0.49 ± 0.12	0.026
MyD88	1.00 ± 0.33	0.28 ± 0.09	0.061
TNF-α	1.00 ± 0.32	1.83 ± 0.28	0.106
IGF1R	1.00 ± 0.09	0.97 ± 0.16	0.866
EGF	1.00 ± 0.27	1.42 ± 0.03	0.314
Occludin	1.00 ± 0.27	0.53 ± 0.15	0.144
Claudin 1	1.00 ± 0.05	1.16 ± 0.03	0.069
Claudin 2	1.00 ± 0.16	1.63 ± 0.13	0.036
ZO-1	1.00 ± 0.28	0.40 ± 0.26	0.182
MUC 1	1.00 ± 0.12	1.40 ± 0.17	0.128
MUC 2	1.00 ± 0.13	1.05 ± 0.27	0.882

^1^ CON, fed with the basal diet; FML, fed with the basal diet supplemented with 10% fermented mulberry leaves. Values are expressed as mean ± standard error, *n* = 6. ^2^ IL-8, interleukin-8; IL-10, interleukin-10; TLR4, Toll-like receptor 4; NFκB-P65, nuclear factor-kappa B; MyD88, myeloid differentiation factor 88; TNF-α, tumor necrosis factor-α; IGF1R, insulin-like growth factor 1 receptor; EGF, epidermal growth factor; ZO-1, zonula occludens-1; MUC 1, mucin 1; MUC 2, mucin 2.

## Data Availability

The data presented in this study are available upon request from the corresponding author.
